# Collaborative working in speech and language therapy for children with DLD—What are parents’ needs?

**DOI:** 10.1111/1460-6984.12951

**Published:** 2023-09-16

**Authors:** Inge S. Klatte, Manon Bloemen, Annemieke de Groot, Tina C. Mantel, Marjolijn Ketelaar, Ellen Gerrits

**Affiliations:** ^1^ Research Center for Healthy and Sustainable Living HU University of Applied Sciences Utrecht Utrecht The Netherlands; ^2^ Institute for Language Sciences Utrecht University Utrecht The Netherlands; ^3^ Department of Education University Medical Centre Utrecht Utrecht The Netherlands; ^4^ Center of Excellence for Rehabilitation Medicine, UMC Utrecht Brain Center University Medical Center Utrecht Utrecht The Netherlands; ^5^ De Hoogstraat Rehabilitation Utrecht The Netherlands

**Keywords:** collaboration, DLD, parents’ needs, speech and language therapy

## Abstract

**Background:**

Collaborative practice between therapists and parents is a key element of family‐centred care and is essential if we want to address family priorities and needs in interventions. However, collaborative practice is challenging for speech and language therapists (SLTs) and parents. To facilitate collaboration, collaborative practices need to be implemented into speech and language therapy for young children with developmental language disorders (DLD) and their families. Actual change and implementation of collaboration in practice will be successful only when it corresponds with patients’ needs, in our case the needs of parents of young children with DLD.

**Aims:**

To explore parents’ needs in their collaboration with SLTs during therapy for their young child with DLD.

**Methods & Procedures:**

Parents of children with (a risk of) DLD in the age of 2–6 years were eligible for participation. We recruited parents via SLTs. Twelve parents of children with DLD participated in semi‐structured interviews about their needs in collaboration with SLTs. We used a phenomenological approach focusing on parents’ lived experiences. We transcribed the interviews verbatim. All interviews were read/listened to and discussed by our parent panel, multiple researchers and the interviewer. Two researchers independently analysed the data using the reflective thematic analysis of Braun and Clarke.

**Outcomes & Results:**

The analysis of the interviews resulted in six themes: (1) knowing what to expect, (2) knowing how to contribute, (3) feeling capable of supporting the child, (4) trusting the therapist, (5) alignment with parents and children's needs, preferences and priorities and (6) time and space for asking questions and sharing information.

**Conclusions & Implications:**

Parents want SLTs to invest time in collaborating with them. Parents need SLTs to empower them to become a collaborative partner and enable them to support their child in daily life. Parents need knowledge about the therapy process and diagnosis and skills in how to support their child's language development. Also, they need emotional support to feel secure enough to support their child, to ask questions to therapists and to bring up their own thoughts and opinions in therapy. Parents’ needs are in line with collaborative working as described in literature, which underlines the importance of implementing collaborative working in speech and language therapy for young children with DLD.

**WHAT THIS PAPER ADDS:**

**What is already known on the subject**

- Several reviews have explored parents’ perspectives on speech and language therapy. Results reveal parents’ experiences with speech and language therapy in general, and parents’ perspectives on specific topics such as shared decision‐making and parents/therapists roles in therapy.

**What this study adds**

- This study adds insights into parents’ needs to ensure collaboration with speech and language therapists (SLTs). Parents of young children with developmental language disorders (DLD) need SLTs to invest time to create optimal collaboration. It is important for parents to have enough knowledge about DLD and the SLT process, skills and confidence in how to support their child and opportunities to share thoughts and questions with SLTs. Our results underline the importance of parents being empowered by SLTs to become a collaborative partner.

**What are the clinical implications of this work?**

- When children are referred to speech and language therapy, parents often venture into an unknown journey. They need support from SLTs to become a collaborative partner in speech and language therapy. Parents need SLTs to invest time in sharing knowledge, skills and power and align therapy to parents’ and child's needs, preferences, priorities and expectations.

## INTRODUCTION

Collaborative working is a key element of family‐centred care and is essential for goal‐setting, planning and implementing interventions that address family priorities and needs (An & Palisano, 2014; Kokorelias et al., 2019). Family‐centred care is related to positive outcomes for parents, families, parent–child interaction and children (Dempsey & Keen, 2008; Dunst et al., 2007; King et al., 2004; Kuhlthau et al., 2011). Collaboration implies therapists and parents share knowledge and skills by mutually supportive interactions and a two‐way interaction style (An & Palisano, 2014). Collaborative working between speech and language therapists (SLTs) and parents of children with developmental language disorder (DLD) is essential for several reasons. DLD has a huge impact on parent–child interaction and on child and parent well‐being (Eadie et al., 2018; Jensen de Lopez et al., 2021). Parents are often worried about their children's feelings and development. They experience having a child with DLD as stressful. Parents feel insecure about their own capabilities in supporting their child's language development (Jensen‐de Lopez et al., 2021). It is therefore important that speech and language therapy for children with DLD not only focuses on the child's development and participation, but also on empowering parents (Klatte et al., 2020). Parents are the centre of learning for children. Evidence shows that the amount and quality of interaction between parent and child influences children's language skills (Hirsh‐Pasek et al., 2015; Levickis et al., 2023; Madigan et al., 2019). For parents it is therefore essential to have the capability and confidence in how to interact with their children to stimulate their language development. Furthermore, parents have insight in the child's strengths and challenges in daily life, know what their child likes and dislikes and can explain what kind of activities align with their family lifestyle and routines (Kokorelias et al., 2019).

The importance of collaborative working in speech and language therapy for young children with DLD is recognised in literature (Melvin et al., 2019; O'Toole et al., 2021; Klatte et al., 2020). However collaborative working is challenging for parents and SLTs (Davies et al., 2017; Klatte & Roulstone, 2016; Watts Pappas et al., 2016). Although SLTs have the intention to collaborate with parents, they often use a one‐directional approach: SLTs make therapy decisions and goals, ask parents to conduct assignments with their child at home and teach parents strategies to use in communication with their child often without discussing parents’ needs and wishes (Bakering et al., in prep; Melvin et al., 2023). A gap is present between theory and practice. This gap could be explained by various factors: (1) the complexity of collaboration (Klatte et al., 2020), (2) the history of therapist‐led working in speech and language therapy (Watts Pappas & McLeod et al., 2008), (3) organisational barriers such as time constraints (Klatte & Roulstone, 2016) and (4) conflicting expectations of parents’ and SLTs about their roles in therapy (Davies et al., 2017).

It is essential to facilitate the implementation of collaborative working between SLTs and parents of children with DLD into practice. In 2020 researchers of the network Collaboration for Communication emphasised the need for explicit knowledge about what collaboration entails for SLTs and parents of children with language difficulties (Klatte et al., 2020). Explicit knowledge about how to collaborate with parents facilitates SLTs’ actual use of collaborative practice in their daily work.

In a systematic review based on studies focusing on parental and therapists’ perspectives on collaboration, we formulated explicit strategies for therapists to ensure collaborative working with parents (Klatte et al, 2023). The strategies are organised into five clusters: (1) continuously invest time in your collaboration with parents, (2) be aware of your important role in the collaboration with parents, (3) tailor your approach, (4) get to know the family and (5) empower parents to become a collaborative partner. Our systematic review had a broader scope than DLD and speech and language therapy: it focused on children with developmental difficulties and their parents, and therapists working with these children. The reason for this broader scope was to learn from other healthcare professionals. So, although explicit knowledge about how therapists can optimise collaboration with parents is presently available, having this knowledge does not solve the implementation gap between literature and practice. Furthermore, many other factors can hinder successful implementation, such as conflicting professional beliefs about the intervention or organisational barriers. Also, the proposed intervention, in our case collaborative working, needs to correspond with local patients’ needs (Damschröder et al., 2009). In order to successfully implement collaborative working in our local SLT context, it is important to know if collaborative working matches the needs of parents of young children with DLD.

Several reviews have explored parents’ perspectives on speech and language therapy (Kwok et al., 2021; O'Toole et al., 2021). However, we are not aware of studies focused specifically on the needs of parents of children with DLD regarding collaboration with SLTs. Therefore, the research question of this current study is: What are parents’ needs in their collaboration with SLTs in therapy for their child with DLD in the age of 2–6 years?

## METHOD

### Methodology

We used a phenomenological approach from a constructionism point of view. We were interested in lived experiences of our participants and we valued subjective experiences of each individual (Braun & Clarke, 2022). Within this approach, we repeatedly reflected on the influence of our perspectives on the data collection and analysis. A parent panel existing of six parents of children with DLD contributed to the data collection and analysis. We used the Standards for Reporting Qualitative Research (O'Brien et al., 2014) in reporting this study. The Internal Ethical Review Board for the health care domain of HU University of Applied Sciences Utrecht provided ethical approval to this study (reference number: 111‐000‐2020_Klatte). All participants gave written informed consent.

### Context of the study

This study is part of a larger project that explored how SLTs can optimise collaboration with parents of 2–6 year‐old children with (risk of) DLD in speech and language therapy. This project was conducted by a consortium that included a parent panel and 12 SLTs. The project focused on SLTs working in primary care in the Netherlands. Usual care performed by these SLTs consists of weekly sessions of 30 min per child for an undetermined period of time. Most SLTs work directly with the child and parents are often present (Gerrits et al., 2019).

### Parent panel

**FIGURE 1:**
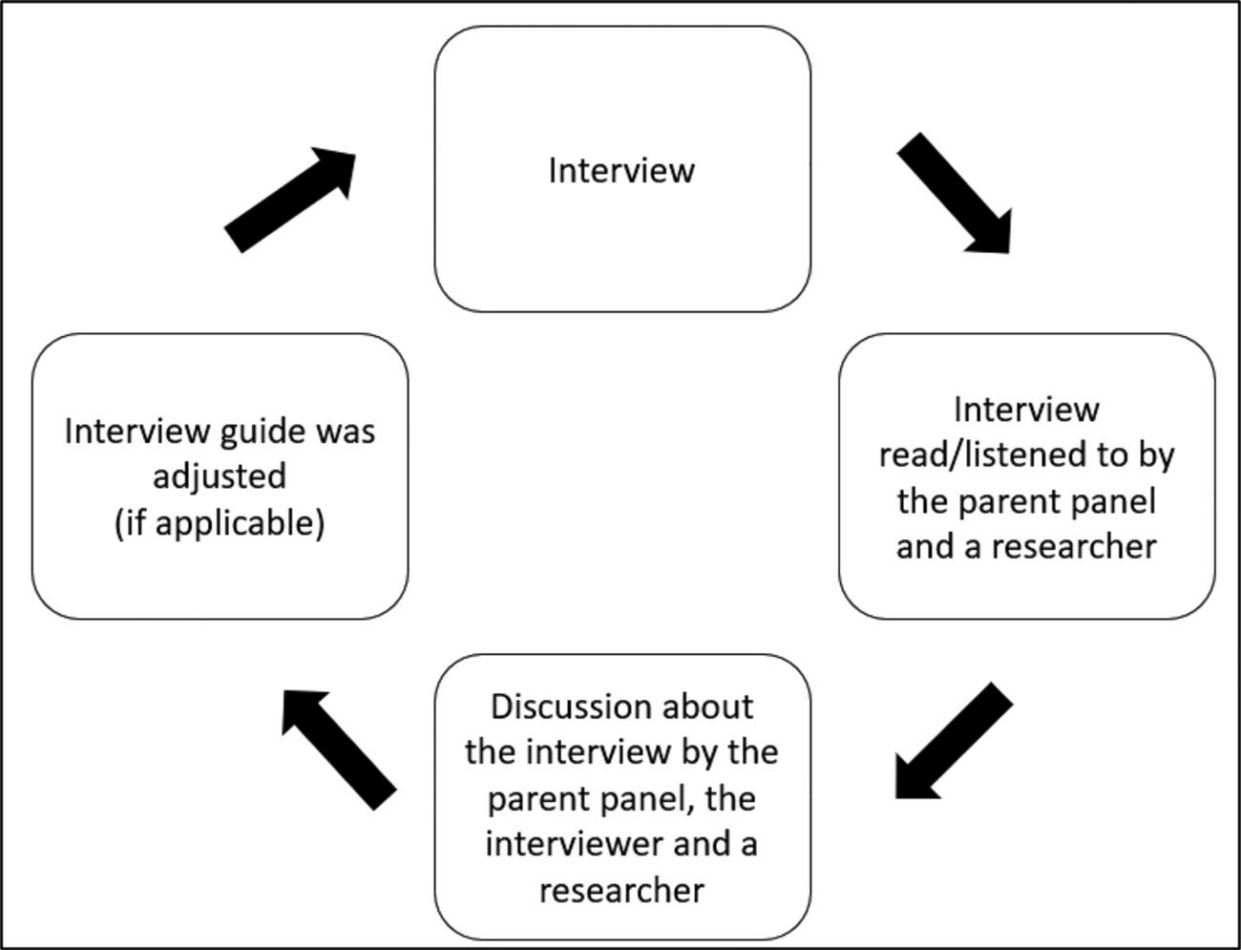
Iterative process of data collection.

We set up a parent panel since we wanted to conduct research about parents with their active involvement in study protocol and data analysis. We recruited parents of children with DLD via a symposium for parents of children with DLD and via social media. Six parents of children with DLD positively responded to our invitation. In order to build a positive collaboration with each panel member, one researcher (I.K.) visited parents and discussed parents’ motivation for being a panel member, and discussed mutual expectations. Also, they discussed the various roles and preferences for their role in the project using the Involvement Matrix (Smits et al., 2020). Parents could choose several roles such as advisor, partner and decision‐maker. In the present study all parents wanted to be advisors in various stages. Parents agreed to listen or read interview manuscripts and discuss their thoughts about the interviews with the research team. One parent also wanted to think along with the interview guide and conduct the interviews together with the interviewer. We were able to fulfil all preferred roles, except for conducting the interviews due to practical reasons. In sum, the parent panel participated as advisors in data collection (Figure 1) and in the familiarisation phase of the data analysis (Table 2).

### Sampling procedures and participants

We included parents of children with DLD 2–6 years of age. In the Netherlands, children are diagnosed with DLD from age 3 onwards by a multidisciplinary team. Children younger than age 3 with low scores on language tests are labelled at risk for DLD and also receive speech and language therapy. Each child had received at least eight speech and language therapy sessions.

Since we were interested in a wide range of lived experiences of parents, we used purposive sampling, focussing on

– Family composition.
– Cultural background.
– SLT's impression of parents’ social‐economic status.
– Child's age.
– Child order in family.
– The amount of sessions the child attended therapy.
– SLT's impression about collaboration between SLT and parent.

We asked the SLTs in our consortium to select three parents of children from their caseload. We purposively selected one parent per SLT. The parents selected were invited by their SLTs and received an information letter about the study. This information letter had been developed earlier in collaboration with the parent panel to ensure parents’ understanding. In total, we invited 18 parents to participate. Seven parents did not want to participate due to several reasons: such as lack of time, frustration with the diagnosis DLD, dealing with the responsibility of caring for their child or not willing to talk about their child's problems. The recruitment process via the SLTs resulted in 11 participants.

Our parent panel advised to recruit an additional participant after the first nine interviews, because data revealed that parents’ experiences about collaboration with the SLT were quite positive. According to the parent panel, this did not reflect parents’ reality. Therefore, we reached out to parents who expressed frustration about collaboration with SLTs via an open invitation on a Dutch Facebook group for parents of children with DLD.

In total, 12 parents participated in an interview. We succeeded in including parents of children between 3 and 6 years old. Characteristics of parents and their children are presented in Table 1.

**TABLE 1:** Participants’ characteristics.

		Parent characteristics		Child characteristics		Collaboration with SLT
	Father/ mother	Family composition	Cultural background	Age	Child's position in family	Poor/ sufficient/ good (SLTs' ratings of collaboration)
P1	Mother	Father, mother and two children	Moroccan	3;2	Youngest	Good
P2	Mother	Father, mother and three children	Turkish	5;0	Youngest	Good
P3	Mother	Father, mother and two children	Dutch and Moroccan	5;6	Oldest	Sufficient
P4	Father	Father and child	Moroccan	4;8	Only child	Poor
P5	Mother	Father, mother and three children	Dutch	3;5	Youngest	Sufficient
P6	Mother	Father, mother and three children	Dutch	5;0	Second	Good
P7	Mother	Father, mother and three children	Dutch and Turkish	6;5	Oldest	Good
P8	Mother	Father, mother and two children	Dutch	5;6	Youngest	Poor
P9	Mother	Father, mother and one child	Sudanese Arabic	5;10	Only child	Good
P10	Mother	Father, mother and two children	Dutch	5;8	Youngest	Unknown^a^
P11	Mother	Father, mother and four children	Dutch	3;0	Youngest	Good
P12	Mother	Mother and three children	Dutch and Spanish	5;9	Youngest	Poor

^a^ This parent was recruited via our parent panel; SLTs’ impression about the collaboration with this parent is therefore lacking.

Abbreviation: SLT, speech and language therapist.

### Data collection

We collected data through semi‐structured interviews which enabled participants to provide in‐depth responses about their needs. Because we wanted to avoid socially accepted answers from parents when talking to SLTs, being SLTs ourselves, the interviewer was a researcher and lecturer within the field of social work. The participants did not know the interviewer prior to the study.

The interviewer followed the interview guide (Appendix A) and also used a visual tool, developed together with the parent panel and an experienced co‐design researcher. The visual tool showed the speech and language therapy process in chronological order. This approach supported parents to feel comfortable in conversing with the interviewer and helped them to express their thoughts. The interviewer used the topics to probe parents’ barriers, facilitators, needs, wishes and expectations regarding their collaboration with the SLT.

The first three interviews took place face to face at parents’ homes. Due to COVID‐19 restrictions, we conducted the other nine interviews online using MS Teams. We audio recorded the interviews and made field notes. The interviews lasted between 23 and 65 min (mean time was 52 min). In order to protect participants’ anonymity, we pseudonymised audio files and transcripts by changing or beeping names. The secured software application ‘SURFdrive’ was used to safely share files with the parent panel. Prior to the study, members of the parent panel had signed a privacy notice.

During data collection we followed an iterative approach: each interview was read/listened to and discussed by the parent panel, researchers (I.K. and/or A.G.) and the interviewer (Figure 1). The aim of these discussions was to share perspectives and interpretations of the interview. This approach is in line with investigator triangulation, which enhanced credibility of our findings. The discussions resulted in small changes in the interview guide. For example, after the first two interviews, the interviewer expressed that it was challenging to talk about collaboration specifically, instead of talking about speech and language therapy in general. Therefore, we decided to add an extra question to the interview guide to explicitly ask parents how they construe collaboration.

### Analysis

We conducted a reflexive thematic analysis by following the six phases as suggested by Braun and Clarke (2022) (Table 2). The steps were independently conducted by multiple researchers and discussed afterwards, to enhance credibility of our findings. We used ATLAS.ti 9 Windows (ATLAS.ti Scientific Software Development GmbH, 2022) for data management.

**TABLE 2:** Analysis of the data.

Phase	Process and persons involved
Familiarising yourself with the data set	This process started during data collection. All interviews were read/listened to and discussed by the parent panel, multiple researchers I.K. and A.G. or T.M.) and the interviewer. Interviews were transcribed verbally.
Coding	Three researchers (I.K., A.G. and T.M.) independently coded and discussed the first interview to gain richer insights into the data. Next, an iterative approach followed: two researchers (I.K. and A.G. or T.M.) independently coded the following interview and discussed their coding prior to the next interview. This phase resulted in 863 initial codes, which are codes close to the data.
Generating initial themes	The same researchers (I.K., A.G. and T.M.) generated initial themes during a face‐to‐face meeting illustrated in a mind map. In preparation of this meeting, they reread the codebook and wrote down their thoughts about initial themes.
Developing and reviewing themes	All researchers discussed the initial themes: some themes were merged, others were split into new (sub) themes. Afterwards, two researchers (I.K. and A.G.) assessed how well the themes matched with the data by returning to the data: reread the interviews, the reflective journal and checked initial themes in the codebook. This phase resulted in six themes.
Refining, defining and naming themes	One researcher (I.K.) wrote a synopsis of each theme. The research team discussed this synopsis and reached consensus about the themes and theme names.
Writing up	One researcher (I.K.) wrote the paper with iterations of writing, reading and editing. The research team gave feedback and supported the writing phase. Much attention was paid to improving the transferability of the findings by sharing information about the context of participants (participant characteristics) and study (Dutch context).

### Researchers characteristics and reflexivity

Four researchers (I.K., A.G., T.M. and E.G.) are qualified SLTs and have experience in working with children with DLD (range of work experience: 3–23 years). Only T.M. still works as an SLT in practice, the other three work in SLT research as a junior researcher (A.G.), PhD student (I.K.) and professor (E.G.). One researcher (M.B.) is educated as paediatric physical therapist and clinical health scientist and works as a senior researcher. One researcher (M.K.) is an associate professor in paediatric rehabilitation with a focus on family‐centred services. The experience in conducting qualitative research differ per researcher: from beginner (A.G. and T.M.) to advanced (M.B. and M.K.). The first author (I.K.) has 8 years of experience in conducting qualitative research. Four researchers (I.K., M.B., A.G. and M.K.) are parents themselves. The interviewer is experienced in coaching parents of children with developmental difficulties and has prior interview experience. The parents in the panel were monolingual Dutch. Two parents had children under the age of 10 years, and four parents had children between 10 and 20 years. Two parents had prior experience in partnering in research as advisors. One parent had DLD himself. The research team kept a reflexive journal to help them reflect about how their own perspectives and experiences might influence the research process and results.

## RESULTS

The data analysis resulted into six themes with several subthemes. To provide context to the description and interpretation of the themes, we start with sharing how parents described collaboration. During the interviews, we felt that it was far more challenging for parents to talk about their own needs and about collaboration with SLTs then talking about their children's needs.

### Parents’ description of collaboration

Most parents described collaboration with SLTs as a one‐way approach where therapists inform parents about their decisions and the therapy plan and give instructions to parents about language support at home. Parents’ voice that their contribution to therapy is to practice at home with their child, as mentioned by P9: *‘I receive the instruction: then you have to do this and this. Then I will follow this instruction. Then we will collaborate in helping my child’ (P9)*. Only a few parents described collaboration wherein parents discuss therapy goals with the SLT, think along with therapy decisions and evaluate the therapy together with the SLT. As P6 described: *‘I think collaboration is that you look at the child together to check if it is still pleasant for the child. How do you experience it, how does the other experience it? So that you really work together’ (P6)*.

The themes and subthemes derived from the interviews are described next, using quotes of parents to illustrate the findings.

### Theme 1: Knowing what to expect

When parents started with speech and language therapy for their child, they often entered an unfamiliar world. Parents expressed feelings of insecurity when attending therapy for the first time, since they did not know why their child was experiencing difficulties. Some explained that having little knowledge on language development and why their child was experiencing difficulties resulted in losing control and feeling dependent of the therapist, as illustrated by P1: *‘Tell me what to do, I don't know it anymore’*. The subthemes are clarity about their child's language difficulties and future perspectives, clarity about the SLT process and clarity about the child's progress. Although all parents expressed the importance of receiving information, some felt overloaded by the amount of information.

– *Clarity about the child's language difficulties and future perspectives*

Most parents started speech and language therapy with worries about their child's development and insecurity about the severity of the problems and the prognosis. Some feel somewhat lost, since they did not know if and why their child's language was lagging behind. Most of them had never heard of DLD before. They wanted to have more control over the situation by learning what normal language development entails, what their child's language difficulties were and what they could expect from the future. Questions like *‘is it possible to get over DLD?’* were raised by several parents. It differs from how parents experienced their child getting a diagnosis. Some parents feel overwhelmed and lost, some did not want their child to have a label, and others feel relieved when it became clear that their child had DLD. *‘She made us aware of DLD, which I appreciated, because I had never heard about it. She gave us a leaflet. And I have looked for information primarily on the internet. It was all new to me. I'm happy that she thought of DLD so it is clear now. […] I know now why he has difficulty with language and that this is persistent. So I can take this into account with certain things*’ *(P8)*.
  Knowledge about the disorder, having examples of other children with DLD, knowing what they can do to help their child and knowing what to expect from the future can help parents to get more control over the situation and let them feel less dependent of the SLT.

– *Clarity about the SLT process*

Most parents were unfamiliar with speech and language therapy prior to their first appointment and did not know what to expect when entering the room. Parents expressed the importance of SLTs sharing information about the SLT process, such as information about the diagnostic phase, frequency and duration of therapy sessions. *‘Sometimes I wonder how long this [speech and language therapy] will take? Because sometimes you see slightly older children and then I wonder, have they been coming here for a long time or do they come for something else? So you do ask yourself, how long do they go to therapy?’ (P3)*.
One parent shared that she was shocked by the fact that her child got tested without her knowing beforehand. She underlined the importance of preparing parents and explaining to parents what kind of test results and implications one can expect. *‘But at a certain time I was overwhelmed that she [SLT] tested him [child]. She said: “well, today we will do an assessment”. I thought: “Okay”. And then she tested him and afterwards she explained why and I was actually slightly startled. […] And a conclusion was made based on these tests which overwhelmed me. I think that the preparation is important, right? That I know: “Okay he will be tested today”. And what you can expect as a parent’ (P1)*.
  Parents also underlined the importance of understanding the added value of speech and language therapy for their child, including specific approaches or materials. One parent called speech and language therapy half an hour of playtime. Although some parents questioned the SLTs’ approach, they did not raise these questions during therapy. As illustrated by P4: *‘the SLT is the expert, she knows what she does. As long as my child shows progress, it is OK’*.

– *Clarity about the child's progress*

The importance of having clarity about the child's progress has been mentioned regularly by parents. Some parents mentioned that they really appreciated it when the therapist took the time to frequently discuss the child's progress. Others shared that they would have liked to have more time to discuss therapy progress with the SLT, based on SLTs’ and parents’ point of view. *‘Well I think a kind of periodical conversation with the SLT. Just having a little bit of time to talk about the current situation, what is the perspective. Are we making progress? Is this the right way? Just to take some time for that. That would make a huge difference’ (P6)*.

### Theme 2: Knowing how to contribute

Almost all parents feel the desire to support their child's development and participation in daily life. Receiving advice from SLTs and observing SLTs during therapy sessions can support parents in helping their child. Some parents expressed the need to practice with their child during therapy sessions, discuss homework assignments and check with the SLT if they perform the assignments correctly. Also, parents would like to have skills in adjusting their language to their child's language level. ‘*I take care that the SLT supports me as well. I have to pay attention. If the SLT spends thirty minutes only on my daughter and then says “bye”. I don't like that. It is important to know what you [the SLT] did with my daughter. What do I need to do to help her*?’ *(P9)*.

### Theme 3: Feeling capable of supporting their child

Parents shared that having a child with language difficulties can have quite an impact on their feelings and well‐being. Parents shared emotions like anxiety, guilt or insecurity. They questioned if they have done something wrong and lost confidence in supporting their child in its development. They need to be reassured by the SLT that they are capable of supporting their child. Also, parents expressed that it is important to know and feel that they are of added value to the expertise of the SLT.

“You [the SLT] really are a coach for the parent and it is just so nice not to be looked at as if it is your fault. You know, as parents we don't know. We are all lay persons. Where does it [DLD] come from? We don't know. So you start to ask yourself questions. And when there is an SLT in front of you who comforts you and gives you confidence. That is just really nice.” P11

### Theme 4: Trust

In order to collaborate with SLTs and be willing to share feelings and thoughts, it is essential for parents to trust the SLT. Parents need to believe that they will be supported by SLTs’ to their best ability. This importance is illustrated by P10 who invests extra traveling time to see a SLT that she trusts: ‘*I called her and said: “Do you have space for [name child] by any chance?” “Yes”, she said, “but you have to be aware of the traveling distance”. I said, “so be it, but I come to you because you are the only one who shows progression with him. We know what to expect from you, so I want to come to you”. So now we travel one hour per week for 30 minutes of speech and language therapy’ (P10)*.

According to parents, trust in SLTs can be influenced by SLTs’ expertise, commitment, empathy towards parents and rapport between parents and SLTs. This is explained in the following subthemes.

– *Expertise*

Parents attend therapy to consult an expert who can help their child in its language development. Therefore, they find it very important that SLTs outline the therapy process and formulate goals, develop a therapy plan and communicate with other relevant professionals. Also, parents find it important that SLTs are honest about the extent of their expertise and introduce parents to other professionals when relevant. One participant explained the need of coming to an expert as follows:
  “[I like therapists to] outline the therapy process. That you [SLT] say this is how are going to do it, based on your professional expertise. I really appreciate our SLT for doing so. She has her own ideas. I know that she is still following courses. It is good to keep up. I really find it important to know that”. P6

– *Commitment*

Parents shared positive experiences with SLTs who were committed with their child and were willing to invest more time and energy in them and their child when necessary. For example, by being approachable outside regular sessions or by checking with parents how they are doing when therapy has finished. ‘*She just calmly explains it and says if you have questions, call me. If I am worried about something, I send her a text message and she calls me the moment she has time. So she is very committed, just a short line between us and that is very pleasant’. (P10)*.

– *Empathy*

For parents it is crucial that SLTs ask parents about how they are doing, how DLD is impacting them and acknowledge their feelings, challenges and worries. Parents describe this as SLTs being empathic, which influences parents openness and willingness to share thoughts. ‘*That you have an hour to pour out your heart for just a while. Because you have to deal with a lot as a parent. I have underestimated that. To be honest, I underestimated it hugely. And to have more space for that… As a parent, or at least, I become uncertain now and then. Do I offer him enough? […] It is not only about the child, but that you [the SLT] can also reassure parents and give them advice’. (P1)*.

– *Rapport*

Having rapport with the SLT was underlined by almost all parents as prerequisite of being able to work together and to trust the therapist. A lack of rapport was for some parents the reason to switch to another therapist.
  “Of course, it is very important that you ‘click’ [with an SLT]. That is valid for any type of health care, rapport comes first. If you don't have that, together you won't be able to proceed”. P11

### Theme 5: Alignment

For parents’ willingness to collaborate with an SLT, alignment is essential. When SLTs align therapy to parents’ and children's situation, parents do not feel like a number, they feel taken seriously and therapy feels relevant for them. The subthemes are align approach to parents’ and children's (changing) needs, preferences and priorities, home assignments and language level.

– *Align approach with children's (changing) needs, preferences and priorities*

Parents emphasised the importance of the happiness and safety of their child during therapy and that the child is willing to participate. Therefore, therapists should align the therapy approach with their child's needs, preferences and priorities. Also, parents expect SLTs to use an holistic view and also focusses on their children's capacities in therapy sessions. ‘*I have made it very clear that [name child] has to have fun. Of course, he gets older and it has to be a bit serious. I don't want to bring him to speech and language therapy crying’ (P2)*.

– *Align approach with parents’ (changing) needs, preferences and priorities*

Parents would like therapists to align with parent's needs, expectations and preferences. Some parents prefer that therapists start directly with assessments and others would like to be prepared before the child gets tested. Some parents prefer to receive a lot of information at the start of therapy, others feel overwhelmed with the amount of information they received. Most parents prefer therapists to play with the child, but some prefer a more firm and direct style. Parents underline the importance of therapists being flexible and adjust their approach when their needs or priorities change over time. For example, P1 expressed that she was happy that the therapist took the lead at the start of the therapy process, however, she would have liked to have a say later on. ‘*She took over when it was necessary, because I was completely lost and I thought: “I don't know what else is going to happen”. And at the moment, she is still deciding what needs to be done in therapy. She is experienced, she is the specialist. But at certain moments I would have liked to have the opportunity to share what I would like to do during a therapy session. “I would like to do this, or I would like to discuss that”. So that I am in control and she does not have to be in the lead all the time’. (P1)*.

– *Align home assignments with parents’ opportunities and preferences*

Aligning home assignments with parents’ opportunities and preferences supports parents to collaborate with SLTs. Parents often feel guilty when they had not practiced with their child. To check with parents if they are able to do assignments or check why parents were not able to perform the activities will support parents to share information about their opportunities and preferences. ‘*Sometimes there is no time to do these things. Sometimes I receive a piece of paper [home assignment] and I have to do that with her [daughter]. This week I don't have any time. I have lots to do, homework and cleaning. And suddenly the week has ended and I have to go to the SLT again. I did not do anything and I feel a bit bad. I don't like that about myself. That's no collaboration. But sometimes parents are busy, cannot help’ (P12)*.

– *Align language level with parents’ level of understanding*

Parents would like SLTs to align their language level to parents’ level of understanding, to enable parents to understand what is going on with their child, what the SLT is doing and why, and how they can contribute to therapy. It is important to explain test results in simple language so that parents are able to explain their child's difficulties in their own words. As P3 expressed, she would have liked to be able to follow conversations about her child's language difficulties. *Parent*: ‘*Well, there was a test result showing that she had made progress on some things and did not show progress on other things. The SLT needed to work on those things. That was what she discussed with me. I don't have the numbers explaining the results. And I don't know if I have to know this just like that, because it is all described in SLT language…’*
*Interviewer*: ‘*Do you want to understand that? Know a bit more about it?’*
*Parent*: ‘*Yes, I don't mind, I would like that. Then I also understand it during a conversation when the school is discussing it, they also use this terminology. And then I think, okay what is 70 and what is 75, is that not good?’*

### Theme 6: Time and space

Almost all parents would like to have more time to discuss their thoughts with and questions with the SLT. Most SLTs work with the child for most of the session time. Only during the last minutes, they discuss home assignments with parents. Most parents prefer time alone with the SLT to discuss their thoughts and ask questions. However, not only having time, also creating space was emphasised. Parents need to feel that their input and questions are appreciated. So, SLTs should invest time and create space for parents to ask questions and to share information, which are the subthemes described next. ‘*I think just giving more space. Even if it is asking at the start “How does it go? Do you want to talk about something?” I think that already helps a lot and I can say “It goes well and I would like to discuss this. Or it doesn't go well”*’ *(P1)*.

– To ask questions

Parents indicated that they have questions, but did not experience time and space to ask these questions, for a variety of reasons. Some felt uncomfortable to ask questions when session time has ended and use someone else's time. Some explained that they did not want to question the therapists’ approach, since the SLT is the expert. Others expressed that they did not ask their questions, since they know that the SLT is very busy. ‘*Since I already know that some SLTs are ill and that they are busy closing another SLT practice. Yes, I always think I cannot really express myself, because they are quite busy already’ (P3)*.

– To share information

Parents would like to share thoughts about several topics, such as their view on their children's progress, what their child's preferences are and what they think that needs to be prioritised in therapy. Although not all parents saw themselves as important partners in their child's therapy, some parents did express to have a lot of useful knowledge about their child. These parents wondered why there was so little time spent on discussing this information. ‘*You [the parent] see the children a lot more than that half hour when the SLT sees the children. So yes, you [the SLT] can profit quite a lot when you start a conversation with parents’ (P6)*.

## DISCUSSION

Exploring the needs of parents of young children with DLD in their collaboration with SLTs resulted in six themes: knowing what to expect, knowing how to contribute, feeling capable of supporting the child, trusting the therapist, alignment, time and space for asking questions and sharing information.

The findings show that parents of young children with DLD want SLTs to invest time in collaborating with them. Speech and language therapy is often an unfamiliar world for parents and most parents have never heard of DLD before. Parents feel often insecure about the difficulties their child is experiencing and how they can support their child's language development. Parents rely on SLTs’ expertise and experience and may even feel dependent on SLTs. These feelings of dependence can be caused by parents’ lack of knowledge about DLD and the speech and language therapy process and lack of skills in how to support their child's language development.

The expressed parental needs in this study relate to empowerment. Empowerment is defined as the combination of ability, motivation and power opportunities (Fumagalli et al., 2015). Ability links to skills and knowledge, which relate to the themes knowing what to expect and how to contribute. Motivation links to attitude and self‐awareness, which relate to feeling capable of supporting their child and trusting the therapist. Power opportunities relate to the theme having time and space for parents to ask questions and share their thoughts. The level of empowerment of parents will differ per person and can be either granted by the professional or acquired by the parent (Fumagalli et al., 2015), which links to the theme alignment. Parents’ empowerment is seen as a key principle of collaboration (An & Palisano, 2014; Klatte et al., 2020): only empowered parents can act as collaborative partners and join shared decision‐making. Empowerment give parents the opportunity to decide if and how they want to participate in goal‐setting, shared implementation and shared evaluation.

The way parents describe collaboration shows a discrepancy in power between parents and SLTs: the SLT takes the lead and parents follow the SLTs’ instructions and contribute by performing activities at home. This links with an advocacy role and fits with results of other studies that focus on parents’ perspectives on their own role in speech and language therapy (Watts Pappas et al., 2016; Davies et al., 2017; Lyons et al., 2010). When asked to express parents’ own needs about collaboration with the SLT, parents struggled with verbalising this. We felt that parents were not used to talking about their own needs instead of their child's needs in the context of speech and language therapy. Parents’ description of collaboration links with a therapist‐led approach wherein a one‐way communication style is used. The reason that parents describe collaboration as therapist led might be caused by the therapist‐led approaches that are being used in practice (Bakering et al., in prep).

## CLINICAL IMPLICATIONS AND FUTURE DIRECTIONS

Our findings show that the needs of parents of young children with DLD regarding collaboration are in line with described strategies about how therapists can collaborate with parents of young children with developmental disorders (Klatte et al., 2023). These strategies focus for example on empowering parents to become a collaborative partner by ensuring parents’ understanding of the therapy process and diagnosis, giving parents emotional support and giving parents the ability and opportunity to bring up things and ask questions. This match between parents’ needs found in this study and strategies regarding collaborative working underlines the importance of implementing collaborative working between SLTs and parents of young children with DLD in speech and language therapy. In our case, implementation means that SLTs use the described collaborative strategies when working with children with DLD and their parents and, by doing that, empower parents to become collaborative partners. Using collaborative strategies requires a change in SLTs’ way of working with children with DLD and their families: away from therapist‐led and child‐centred, towards family‐centred care using collaborative working. Changing behaviour is challenging and is influenced by one's capability, motivation and opportunity (Michie et al., 2014). Behaviour change requires a well thought out plan based on local facilitators and barriers. Therefore, research into SLTs’ barriers and facilitators in working collaboratively with parents of young children with DLD is necessary to get more insight into suitable implementation strategies.

## STRENGTHS AND LIMITATIONS

A limitation of this study is that we have interviewed parents who were willing to talk about their experiences and needs. Some parents who were invited were unwilling to participate because of frustration with the diagnosis or not wanting to talk about their child's difficulties. Talking to these parents could have added useful insights to current findings. There might be a blind spot in our findings and in literature on parental needs, since parents who are frustrated with therapy or diagnosis might not be willing to participate in research studies in general. In our study, inviting parents to participate via the therapists of their child might have caused the unwillingness. For future research we suggest to recruit parents via other parents, instead of SLTs. The strengths of our recruitment approach is that we were able to reach parents with diverse cultural backgrounds. Although our research question focuses on 2‐ to 6‐year‐old children, no parents of 2‐year‐old children participated in this study. However, we do not expect that having a 2‐year‐old child instead of a 3‐year‐old child would have had influenced parents’ experiences.

We feel that the active involvement of parents in a parent panel was of great value to our data collection and analysis. Discussing the transcripts with parents stimulated us as SLT researchers to step away from our own perspectives and to learn from parent perspectives when interpreting the data. This approach added to the credibility of the findings. One parent suggested to participate in the interviews with parents as co‐interviewer which could have had a positive influence on the data collection: participants might have responded differently to a parent than to a professional. We were not able to organise this due to practical reasons such as time and money. For future research, it is important to cover flexibility in how to conduct a research project in future grant applications.

We asked a non‐SLT to conduct the interviews with parents, since we expected parents to be more open about their collaboration with SLTs when they could talk to someone outside the speech and language therapy profession. The downside of this decision was that the interviewer was not fully aware of the speech and language therapy process and might have missed some relevant cues to probe on during the interview. We tried to overcome this by discussing each interview with her prior to the next one, and hence supporting the interviewer with relevant knowledge about the SLT process.

## CONCLUSION

Parents want SLTs to invest time in collaborating with them. Parents need SLTs to empower them to become a collaborative partner and enable them to support their child in daily life. Parents need knowledge about the therapy process and diagnosis, skills in how to support their child's language development and emotional support to feel secure enough to support their child, ask questions to therapists, and bring up their own thoughts and opinions in therapy. Parents’ needs are in line with collaborative working as described in literature, which underlines the importance of implementing collaborative working in speech and language therapy for young children with DLD.

## CONFLICT OF INTEREST STATEMENT

The authors declare no conflicts of interest.

## Data Availability

The data that support the findings of this study are available on request from the corresponding author. The data are not publicly available due to privacy or ethical restrictions.

## INTERVIEW GUIDE

**Table:**

Interview guide
First question:
Could you tell me what your family looks like? (Use playmobile puppets to visualise the family members) Second question:
Could you tell me why you and your child started with speech and language therapy? Topics to probe when discussing the time path: – Barriers in collaborating with the SLT. – Facilitators in collaborating with the SLT. – Parents needs and wishes regarding the collaboration with the SLT. – Parents’ expectations regarding speech and language therapy and their collaboration with the SLT.
Final question:
Is there anything you have missed or like to add?

Abbreviation: SLT, speech and language therapist.
